# Comparative effectiveness of echinocandins and liposomal amphotericin B for fluconazole-resistant *Candida parapsilosis* bloodstream infections

**DOI:** 10.1128/aac.00355-25

**Published:** 2025-09-30

**Authors:** Antonio Vena, Claudia Bartalucci, Marco Muccio, Giusy Tiseo, Patricia Muñoz, Mario Cesaretti, Vincenzo Di Pilato, Anna Marchese, Ramona Barbieri, Arianna Forniti, Daniele Roberto Giacobbe, Alessandro Limongelli, Antonella Lupetti, Malgorzata Mikulska, Jon Salmanton-García, Ana Soriano Martín, Lucia Taramasso, Maricela Valerio, Pilar Escribano, Jesus Guinea, Emilio Bouza, Marco Falcone, Matteo Bassetti, On behalf of ESCMID Fungal Infection Study Group (EFISG)

**Affiliations:** 1 Infectious Diseases Unit, Policlinico San Martino Hospital – IRCCS 9246 , Genoa, Italy; 2 Department of Health Sciences (DISSAL), University of Genoa 9302, Genoa, Italy; 3 Infectious Diseases Unit, Department of Clinical and Experimental Medicine, Azienda Ospedaliero Universitaria Pisana 9257, Pisa, Italy; 4 Clinical Microbiology and Infectious Diseases, Hospital General Universitario Gregorio Marañón 16483, Madrid, Spain; 5 Instituto de Investigación Sanitaria Hospital Gregorio Marañón 624712, Madrid, Spain; 6 CIBER Enfermedades Respiratorias-CIBERES (CB06/06/0058) 568067 , Madrid, Spain; 7 Medicine Department, School of Medicine, Universidad Complutense de Madrid 16734, Madrid, Spain; 8 Department of Surgical Sciences and Integrated Diagnostics (DISC), University of Genoa 9302, Genoa, Italy; 9 Microbiology Unit, IRCCS Ospedale Policlinico San Martino 9246 , Genoa, Italy; 10 Department of Translational Research and New Technologies in Medicine and Surgery, University of Pisa 9310, Pisa, Italy; 11 Institute of Translational Research, Cologne Excellence Cluster on Cellular Stress Responses in Aging-Associated Diseases (CECAD), Faculty of Medicine and University Hospital Cologne, University of Cologne, Cologne, Germany; 12 Department I of Internal Medicine, Center for Integrated Oncology Aachen Bonn Cologne Duesseldorf (CIO ABCD), University of Cologne 14309, Cologne, Germany; 13 Department I of Internal Medicine, European Confederation for Medical Mycology (ECMM) Excellence Center, University of Cologne 61059, Cologne, Germany; 14 German Centre for Infection Research (DZIF), Partner Site Bonn-Cologne, Cologne, Germany; 15 Faculty of Health Sciences - HM Hospitals, Universidad Camilo José Cela, Madrid, Spain; University Children's Hospital Münster, Münster, Germany

**Keywords:** *Candida* bloodstream infection, *Candida parapsilosis*, fluconazole resistance, echinocandins, liposomal amphotericin B

## Abstract

Current therapeutic options for fluconazole-resistant *Candida parapsilosis* (FLZR-CP) bloodstream infections (BSI) are limited to echinocandins and liposomal amphotericin B (L-AmB). To the best of our knowledge, no real-world comparative effectiveness studies have assessed these agents. This study aimed to compare the effectiveness of echinocandins and L-AmB for the treatment of FLZR-CP BSI. This retrospective, observational study was conducted in two hospitals in Italy between January 2018 and December 2022. Eligible patients were adults (≥18 years old) with microbiologically confirmed FLZR-CP BSI who received targeted therapy with either echinocandins or L-AmB. Patients were matched (2:1) based on age, Charlson comorbidity index, presence of sepsis or septic shock, time to appropriate antifungal therapy (≤48 hours or > 48 hours from diagnosis), and infection source. A total of 63 patients were included (42 in the echinocandin group and 21 in the L-AmB group). In Cox regression, targeted therapy with echinocandins was not associated with increased mortality (adjusted hazard ratio 1.40; 95% confidence interval [CI] 0.33–5.92, *P* = 0.645). An exploratory sensitivity analysis including patients who did not receive source control yielded consistent results (*P* = 0.491). Furthermore, in the multivariable regression analysis, echinocandin therapy was not associated with an increased risk of persistent fungemia (adjusted odds ratio 1.61: 95% CI 0.43–5.99, *P* = 0.476). Treatment with echinocandins and L-AmB resulted in similar 30-day mortality and persistent fungemia rates in patients with FLZR-CP BSI. These findings confirm that echinocandins are a viable treatment option for *C. parapsilosis* BSI, even for patients with fluconazole-resistant strains.

## INTRODUCTION

Fluconazole-resistant *Candida parapsilosis* (FLZR-CP) has increasingly been recognized as a concerning antifungal-resistant pathogen that poses a significant threat to hospitalized patients in many countries (1–3). Although traditionally considered less virulent than other *Candida* species (4), FLZR-CP has proven to be a concerning pathogen (1, 2), capable of causing persistent nosocomial outbreaks that are difficult to eradicate despite strict infection control measures (5). Infections caused by FLZR-CP are challenging to treat, as therapeutic options are limited to echinocandins and liposomal amphotericin B (L-AmB), with no oral options for step-down therapy.

The latest guidelines (6–8) do not provide specific recommendations for the management of bloodstream infection (BSI) caused by FLZR-CP. Consequently, clinicians often rely on echinocandins, extrapolating recommendations established for fluconazole-susceptible (FLZS) strains to resistant ones (3). However, *C. parapsilosis* exhibits intrinsically higher minimum inhibitory concentrations (MICs) to echinocandins (9, 10), and these agents may not always be appropriate due to their poor penetration into certain tissues, such as the eye or the urinary tract (11). Additionally, there are increasing reports of FLZR-CP isolates that are also resistant to echinocandins (12). L-AmB represents an alternative for the treatment of FLZR-CP infections, supported by *in vitro* susceptibility data and clinical experience (13). However, L-AmB is associated with notable adverse effects, including nephrotoxicity (14), electrolyte abnormalities (15), and high cost (16). Despite the urgent clinical need, there are currently no randomized controlled trials comparing echinocandins and L-AmB for the treatment of FLZR-CP BSI. As such, the optimal therapeutic approach for managing severe infections caused by FLZR-CP remains unknown.

In this study, we conducted a retrospective comparative effectiveness analysis of echinocandins and L-AmB for BSI caused by FLZR-CP.

## MATERIALS AND METHODS

### Study design and patients

This matched, retrospective, observational cohort study was conducted at two hospitals in Italy: San Martino Polyclinic Hospital in Genoa and Azienda Ospedaliera Pisana in Pisa. All consecutive adult inpatients (≥18 years old) with monomicrobial BSI due to FLZR-CP from 1 January 2018 to 31 December 2022 were eligible for inclusion. Patients were allocated to the echinocandin or L-AmB group based on the targeted antifungal therapy prescribed by the treating physicians. Exclusion criteria included (i) isolates demonstrating non-susceptibility to the administered antifungal agent, as defined by the Clinical Laboratory Standards Institute (CLSI) breakpoints (M60, 2nd edition), and (ii) receipt of combination therapy, defined as co-administration of antifungal agents for more than 48 hours. Additionally, episodes of recurrent FLZR-CP BSI were excluded, with only the first episode per patient considered for analysis. The study was approved by the medical ethical committee of each participating center (MICRO.HGUGM.2021-030) and was performed in accordance with the Declaration of Helsinki.

### Procedures

Eligible patients receiving echinocandins or L-AmB were matched in a 2:1 ratio using Mahalanobis (17) distance on age and Charlson Comorbidity Index. Exact matching was applied for (i) time to initiation of appropriate antifungal therapy (≤48 hours vs >48 hours, calculated from FLZR-CP BSI onset), (ii) source of infection (categorized as primary/Central Venous Catheter [CVC]-related, intra-abdominal, or other), and (iii) presence or absence of sepsis or septic shock, which were grouped together. Data collection included demographics, hospital ward at the time of FLZR-CP BSI onset, underlying conditions assessed using the Charlson Comorbidity Index, risk factors for *Candida* BSI, prior antifungal treatments, clinical presentation including sepsis and septic shock, source of FLZR-CP BSI, performance and timing of the source control procedure, diagnostic procedures (e.g., ophthalmological examination and echocardiography), presence of persistent fungemia, and 30-day all-cause mortality.

### Exposure variable

The primary exposure variable was targeted antifungal treatment with either echinocandins or L-AmB for the management of FLZR-CP BSI. Patients receiving sequential therapy were classified into the echinocandin or L-AmB group based on whether they received at least 50% of the total treatment duration with the respective antifungal agent. Any initial antifungal therapy was permitted, provided that subsequent antifungal treatment met the criteria for appropriate targeted therapy.

### Outcomes

The primary outcome of the study was 30-day all-cause mortality. Secondary outcome included the rate of persistent BSI due to FLZR-CP, defined as positive follow-up blood cultures obtained at least 5 days after initiation of the study drug.

### Definitions

An episode of FLZR-CP BSI was defined as at least one peripheral blood culture positive for FLZR-CP in a hospitalized patient exhibiting signs and/or symptoms of infection. The onset of FLZR-CP BSI was determined by the date of collection of the first blood culture yielding the infecting organism. The presence of sepsis or septic shock was documented on the same day as BSI onset (18). Initial antifungal therapy was defined as the first systemic antifungal given after a positive peripheral blood culture and was considered appropriate if FLZR-CP showed *in vitro* susceptibility.

Patients were classified as having primary candidemia if no clear source of infection was identified or if the infection was likely associated with a CVC (19). CVC-related candidemia was defined according to established guidelines (6). The abdomen was considered the source of FLZR-CP BSI if there was evidence of an intra-abdominal infection, with either (i) a positive culture obtained from the intra-abdominal cavity via surgery or needle aspiration or (ii) no other identifiable sources of candidemia (19). Source control measures included CVC removal or invasive procedures such as relief of urinary tract obstruction or drainage of intra-abdominal abscesses, depending on the primary site of infection.

### Microbiological studies

During the entire study period, each participating center adhered to local guidelines recommending the collection of at least two blood samples (approximately 20 mL each for adults) to assess each suspected episode of BSI. Blood from each extraction was evenly distributed between aerobic and anaerobic culture bottles. *Candida* species identification and *in vitro* antifungal activity were evaluated at the participating hospitals using standard local methods. Identification of *Candida* species was performed using classical phenotypic methods in conjunction with matrix-assisted laser desorption ionization-time of flight mass spectrometry across all centers. Antifungal susceptibility testing was conducted using a commercial microdilution method (Sensititre YeastOne, ThermoFisher Scientific Inc., Waltham, MA, USA), following the manufacturer’s instructions. Interpretive breakpoints were based on the CLSI performance standards for antifungal susceptibility testing of yeasts (M60, 2nd edition). Fluconazole resistance was defined as a MIC > 4 µg/mL, in accordance with the CLSI reference method for broth dilution antifungal susceptibility testing of yeast (4th edition, CLSI document M27-A4; 2017).

### Statistical analysis

Continuous variables were compared between the two groups using the Wilcoxon rank-sum test, while categorical variables were analyzed using the Chi-squared or Fisher’s exact test, as appropriate. All-cause mortality at 30 days from the onset of FLZR-CP BSI was graphically summarized using Kaplan-Meier curves, and differences between patients treated with targeted echinocandins or L-AmB were assessed using the log-rank test.

Risk factors for mortality were analyzed using Cox regression models following Rubin’s multiple imputation of missing values for categorical variables (20) and median imputation for numerical variables. Variables with a *P*-value < 0.1 in univariable Cox analysis or in the initial between-group comparison were included in a multivariable model and further selected for inclusion in the final multivariable Cox regression model using a backward stepwise procedure. Given the primary objective of the study, the variable “targeted therapy with either echinocandins or L-AmB” was retained in the model regardless of stepwise selection. Variables included in the final multivariable model were also incorporated into (i) an additional multivariable Cox regression model incorporating the study center as a shared frailty factor (21); (ii) a landmark analysis, using the 5th day after FLZR-CP BSI onset to mitigate potential confounding from early mortality; and (iii) a separate shared frailty model accounting for matched patient clusters. To further explore the impact of targeted therapy on 30-day all-cause mortality, we conducted a first exploratory sensitivity analysis including a small subgroup of patients (*n* = 11) who did not undergo adequate source control. A second sensitivity analysis was also performed based on the initial antifungal agent administered. Risk factors for persistent FLZR-CP BSI were analyzed using a logistic regression model. Following imputation of missing values, variables with a *P*-value < 0.1 in univariable analysis or in the initial between-group comparison were included in a multivariable backward stepwise logistic model. The variables selected in this process were then incorporated into the final multivariable logistic regression model, as well as in an additional model adjusted for clustering within matched sets. Given the study’s objective, the variable “targeted therapy with either echinocandins or L-AmB” was included in the model regardless of stepwise selection. All statistical analyzes were conducted using SAS software (version 9.4, SAS Institute Inc., Cary, NC, USA). A *P*-value < 0.05 was considered statistically significant.

## RESULTS

During the study period, 196 patients received targeted therapy with echinocandins, and 22 were treated with L-AmB for FLZR-CP BSI. Of these, one L-AmB-treated patient could not be adequately matched based on the predefined criteria and was excluded. As a result, 63 patients were included in the final matched cohort: 42 treated with echinocandins and 21 with L-AmB. No patients were excluded due to non-susceptibility to the administered antifungal agent or receipt of combination therapy.

### Baseline clinical characteristics

The median age was 63 years (interquartile range [IQR] 58–72), and 33 patients (52.4%) were male. Cardiovascular disease was present in 27 patients (42.8%), while 15 (23.8%) had a solid organ tumor. Features of patients and crude outcomes according to type of targeted therapy received are shown in Table 1. Baseline characteristics were comparable between the two groups, with the exception of a higher prevalence of liver disease (four out of 21 [19.0%] vs one out of 42 [2.4%], *P* = 0.0387), prior intra-abdominal surgery (11 out of 21 [52.4%] vs eight out of 41 [19.5%], *P* = 0.0079) and prior antifungal exposure (13 out of 21 [61.9%] vs six out of 41 [14.6%], *P* = 0.0001) in the L-AmB group. In the echinocandin group, caspofungin was the most frequently used agent (38 patients; 90.7%), followed by micafungin and anidulafungin (two patients each; 4.7%). All patients received standard echinocandin dosing regimens, although two patients with infective endocarditis received a higher maintenance dose of caspofungin (70 mg daily). All patients receiving L-AmB were treated with a dosage of 3 to 5 mg/kg IV once daily.

**TABLE 1:** Baseline demographics and clinical characteristics of patients in the entire study population and in patients treated with echinocandins and L-AmB^*a*^^,^^*g*^^,^^*h*^

Variables^*b*^	Total *N* = 63	Echinocandins *N* = 42	L-AmB *N* = 21	*P*-value
Age, median [IQR], y	63 (58–72)	64 (59–71)	63 (58–72)	0.9942
Male sex; *n* (%)	33 (52.4)	22 (52.4)	11 (52.4)	1.0000
Hospital ward stay at the time of *C. parapsilosis* BSI; *n* (%)				0.5460^*f*^
Intensive care unit	40 (63.5)	25 (59.5)	15 (71.4)	
Surgical ward	12 (19.1)	8 (19.1)	4 (19.1)	
Internal medicine ward	11 (17.5)	9 (21.4)	2 (9.5)	
Time between hospital admission and first positive BC; median [IQR], d	35 (21–59)	30.5 (19–59)	36 (28–62)	0.2805
Charlson Comorbidity Index; median (IQR)	2 (1–3)	2 (1–3)	2 (1–3)	1.0000
Underlying conditions, n (%)				
Cardiovascular disease	27 (42.8)	19 (45.2)	8 (38.1)	0.5892
Gastrointestinal disease	17 (27.0)	10 (23.8)	7 (33.3)	0.4221
Solid tumor	15 (23.8)	10 (23.8)	5 (23.8)	1.0000
Diabetes mellitus	11 (17.5)	5 (11.9)	6 (28.6)	0.1575^*f*^
Neurological disease	9 (14.3)	7 (16.7)	2 (9.5)	0.7052^*f*^
Chronic kidney disease	8 (12.7)	6 (14.3)	2 (9.5)	0.7079^*f*^
Chronic lung disease	8 (12.7)	6 (14.3)	2 (9.5)	0.7079^*f*^
Chronic liver disease	5 (7.9)	1 (2.4)	4 (19.1)	**0.0387^*f*^**
Solid organ transplantation	3/62 (4.8)	1/41 (2.4)	2/21 (9.5)	0.2628^*f*^
Hematological malignancy	2 (3.2)	1 (2.4)	1 (4.8)	1.0000^*f*^
Risk factors for candidemia, *n* (%)				
Antibiotic therapy^*d*^	58 (92.1)	38 (90.5)	20 (95.2)	0.6570^*f*^
Central venous catheter	48 (76.2)	29 (69.1)	19 (90.5)	0.0598
Surgery (all types)^*e*^	40 (63.5)	26 (61.9)	14 (66.7)	0.7113
Total parenteral nutrition^*d*^	35/60 (58.3)	20/40 (50.0)	15/29 (75.0)	0.0641
Corticosteroid therapy^*d*^	25/62 (40.3)	18/41 (43.9)	7/21 (33.3)	0.4220
Intra-abdominal surgery^*e*^	19/62 (30.7)	8/41 (19.5)	11/21 (52.4)	**0.0079**
Hemodialysis^*e*^	15 (23.8)	8 (19.1)	7 (33.3)	0.2095
Immunosuppressive therapy^*d*^	7/62 (11.3)	3/42 (7.1)	4/20 (20.0)	0.1986^*f*^
Chemotherapy^*e*^	2 (3.2)	1 (2.4)	1 (4.8)	1.0000^*f*^
Previous antifungal treatment, *n* (%)^*d*^	19/62 (30.7)	6/41 (14.6)	13/21 (61.9)	**0.0001**
Septic shock,^*b*^ *n* (%)	13 (20.6)	8 (19.1)	5 (23.8)	0.7451^*f*^
Primary/catheter source of origin (vs abdominal); *n* (%)	57 (90.5)	38 (90.5)	19 (90.5)	1.0000^*f*^
Initial antifungal therapy, *n* (%)				< 0.0001^*f*^
Echinocandins	51 (81.0)	39 (92.9)	12 (57.1)	
L-Amb	9 (14.3)	0 (0.0)	9 (42.9)	
Fluconazole	3 (4.8)	3 (7.1)	0 (0.0)	
Appropriate initial antifungal therapy, *n* (%)	60 (95.2)	39 (92.8)	21 (100)	0.5447
Time between first positive BC and appropriate antifungal therapy,^*b*^ median (IQR), d	2 (1–3)	2 (1–3)	2 (1–3)	0.7158
Source control done; *n* (%)				0.0746^*f*^
Yes	50/61 (82.0)	31/40 (77.5)	19/21 (90.5)	
No	4/61 (6.6)	2/40 (5.0)	2/21 (9.5)	
Not possible	7/61 (11.5)	7/40 (17.5)	0/21 (0.0)	
Time between first positive BCs and source control, median [IQR], d	*n* = 48 3 (1–4)	*n* = 30 3 (1-3)	*n* = 18 3 (2–4)	0.5587
Persistent *C. parapsilosis* BSI; *n* (%)^*c*^	22/60 (36.7)	15/41 (36.6)	7/19 (36.8)	0.5399^*f*^
Diagnostic procedures, *n* (%)				
Echocardiography and echocolordoppler	59 (93.7)	39 (92.9)	20 (95.2)	1.0000^*f*^
Ophthalmologic examination	37 (58.7)	25 (59.5)	12 (57.1)	0.8564
Complications, *n* (%)				
Ocular candidiasis				0.3798^*f*^
No	36/37 (97.3)	25/25 (100.0)	11/12 (91.7)	
Yes	1/37 (2.7)	0/25 (0.0)	1/12 (8.3)	
Endocarditis or thrombophlebitis				0.2532^*f*^
No	53/59 (89.8)	37/39 (94.9)	16/20 (80.0)	
Yes	6/59 (10.2)	2/39 (5.1)	4/20 (20.0)	
Intensive care unit admission	5 (7.9)	3 (7.1)	2 (9.5)	1.0000^*f*^
Need for hemodialysis after *C.parapsilosis* BSI	9/62 (14.5)	5/41 (12.2)	4/21 (19.1)	0.4725^*f*^
30-d all-cause mortality, *n* (%)	15 (23.8)	8 (19.1)	7 (33.3)	0.2095
Time between first positive BCs and death, median [IQR], d	*n* = 23 17 (7–46)	*n* = 13 17 (7–46)	*n* = 10 17 (8–31)	0.8037

^*a*^ The reported *P*-values are from the Wilcoxon rank sum test for continuous variables and Chi-square or Fisher’s exact test for categorical variables; bold values are significant at the selected level of significance (α = 0.05). Where indicated, due to missing values, the analysis is based on the available sample size.

^*b*^ Variable transformed for matching purposes and differs from the categorization presented in this table. Missing values were imputed using the median prior to matching. Only one patient with imputed data was included in the final matched cohort, and thus no missing values remained for this variable after matching.

^*c*^ Follow-up blood cultures were performed in 60/63 patients (95.2%). Among them, 41 have been treated with echinocandins, and 19 have been treated with L-AmB.

^*d*^ Within the prior 30 days.

^*e*^ Within the prior 90 days.

^*f*^ Fisher’s exact test.

^*g*^ Abbreviations: BC: Blood cultures; BSI: bloodstream infection; D: days; IQR: interquartile range; Y: years; FLZR-CP BSI: fluconazole-resistant *C. parapsilosis* bloodstream infection; L-AmB: liposomal amphothericin B.

^*h*^ Empty cells indicate where the *P* value refers to the comparison between the variables listed in the subsequent rows.

### Analysis of the impact of targeted antifungal therapy on the 30-day all-cause mortality rate

**Fig 1:**
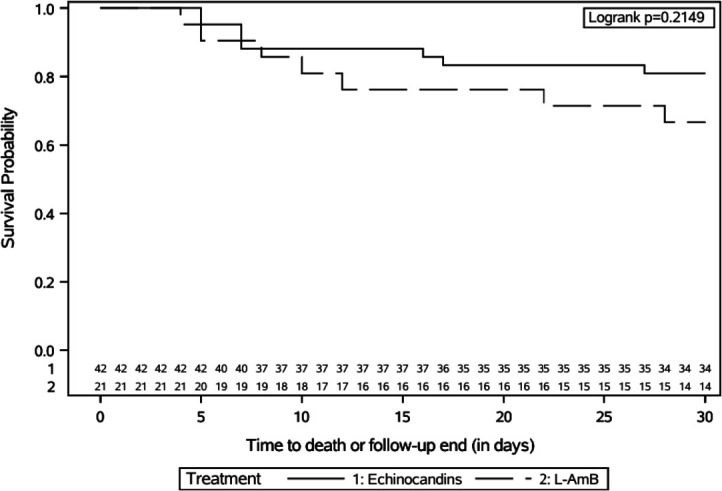
Unadjusted cumulative survival probability distribution up to day 30 in patients with FLZR-CP BSI treated with echinocandins or L-AmB.

**Fig 2:**
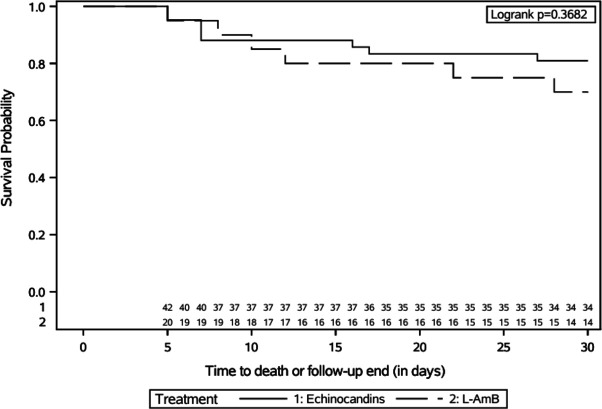
Landmark analysis (5 days) of unadjusted cumulative survival probability distribution up to day 30 in patients with FLZR-CP BSI treated with echinocandins or L-AmB (62 subjects, 14 deaths).

Thirty-day all-cause mortality among patients treated with echinocandins or L-AmB was 19.1% and 33.3%, respectively (*P* = 0.2095). Kaplan-Meier curves for 30-day all-cause mortality did not show significant difference between patients treated with targeted echinocandins or L-AmB (log-rank test, *P* = 0.2149) (Fig. 1). Univariable and multivariable analyses of factors associated with 30-day all-cause mortality are detailed in Table 2. Targeted therapy with echinocandins rather than L-AmB was not found to be associated with 30-day mortality (adjusted hazard ratio [aHR] 1.40; 95% confidence interval [CI] 0.33–5.92, *P* = 0.6452). Including center as shared frailty did not alter the results of the model (Table 3). A landmark analysis, restricted to patients who survived beyond 5 days after the onset of FLZR-CP BSI, yielded similar results (Table 3, Fig. 2). Similarly, the shared frailty model accounting for matched clusters confirmed the primary findings (aHR 0.68; 95% 0.24–1.97, *P* = 0.4777; data not shown). The exploratory sensitivity analysis, including patients who did not undergo adequate source control (*n* = 11), yielded results consistent with those of the overall cohort (Fisher’s test, *P* = 0.4909). (Fig. S1). The second sensitivity analysis, based on initial antifungal therapy, also showed no significant survival difference between groups (log-rank test, *P* = 0.8786; Fig. S2).

**TABLE 2:** Univariable and multivariable with backward selection analyses of factors associated with all-cause 30-day mortality in the study population, after missing imputation^*a*^^,^^*i*^^,^^*j*^

	Univariable		Multivariable		Multivariable backward^*c*^	
Variable	HR (95% CI)	*P*-value	aHR (95% CI)	*P*-value	aHR (95% CI)	*P*-value
Treatment (echinocandin vs L-AmB)	0.54 (0.20–1.48)	0.2292	1.40 (0.33–5.92)	0.6452	0.68 (0.24–1.97)	0.4777
Age, y	1.02 (0.98–1.07)	0.2777				
Male sex vs female sex	1.42 (0.51–4.00)	0.5041				
Time between hospital admission and first positive BC, d	1.00 (0.99–1.01)	0.6073				
Charlson comorbidity index	1.12 (0.90–1.39)	0.2951				
Underlying conditions						
Cardiovascular disease (yes vs no)	0.91 (0.32–2.55)	0.8549				
Chronic kidney disease (yes vs no)	1.08 (0.24–4.77)	0.9221				
Diabetes mellitus (yes vs no)	2.52 (0.86–7.37)	0.0926	4.98 (1.32–18.70)	**0.0175**	4.61 (1.31–16.26)	**0.0176**
Chronic lung disease (yes vs no)	3.41 (1.08–10.77)	**0.0368**	5.66 (1.20–26.60)	**0.0282**	5.65 (1.47–21.72)	**0.0117**
Gastrointestinal disease (yes vs no)	0.98 (0.31–1.09)	0.9779				
Solid tumor (yes vs no)	0.72 (0.20–2.53)	0.6031				
Risk factors for candidemia						
Hemodialysis^*e*^ (yes vs no)	1.27 (0.41–3.99)	0.6814				
Immunosuppressive therapy^*d*^ (yes vs no)	2.48 (0.70–8.80)	0.1606				
Corticosteroid therapy^*d*^ (yes vs no)	1.44 (0.52–3.97)	0.4820				
Antibiotic therapy^*d*^ (yes vs no)						
Central venous catheter (yes vs no)	2.32 (0.52–10.28)	0.2683	1.56 (0.25–9.55)	0.6318	–	
Total parenteral nutrition^*d*^ (yes vs no)	1.63 (0.56–4.77)	0.3716	1.59 (0.39–6.58)	0.5192	–	
Surgery (all types)^*e*^ (yes vs no)	1.17 (0.40–3.42)	0.7744				
Intra-abdominal surgery^*e*^ (yes vs no)	2.08 (0.75–5.73)	0.1581	2.40 (0.49–11.87)	0.2825	–	
Previous antifungal treatment^*d*^ (yes vs no)	2.06 (0.75–5.70)	0.1621	1.40 (0.34–5.76)	0.6411	–	
Time between first positive BC and appropriate antifungal therapy, d	0.78 (0.55–1.10)	0.1543				
Septic shock (yes vs no)	4.40 (1.59–12.22)	**0.0044**	3.64 (0.92–14.48)	0.0664	6.22 (1.96–19.70)	**0.0019**
Primary/catheter source of origin (vs abdominal)	0.37 (0.11–1.32)	0.1257				
Source control done (yes vs no)	0.31 (0.07–1.38)	0.1244	0.10 (0.02–0.66)	**0.0165**	0.12 (0.02–0.67)	**0.0156**
Source control done (not possible vs no)^*b*^						
Time between first positive BCs and source control, d^*f*^	0.96 (0.78–1.19)	0.7248				
Persistent *C. parapsilosis* BSI (yes vs no)^*g*^	0.67 (0.21–2.17)	0.5001				
Endocarditis or thrombophlebitis (yes vs no)^*h*^	1.46 (0.32–6.61)	0.6205				
Need for hemodialysis after *C.parapsilosis* BSI (yes vs no)	4.36 (1.55–12.28)	**0.0053**	1.57 (0.38–6.52)	0.5330	–	

^*a*^ Analyses conducted after multiple imputation.The reported *P*-values are from the Cox regression analysis. Bold values are significant at the selected level of significance (α = 0.05).

^*b*^ Rows marked, as well as the following variables, were excluded from the analysis due to an insufficient number of events: hospital ward stay at the time of *C. parapsilosis* BSI, neurological disease, chronic liver disease, hematological malignancy, solid organ transplantation, chemotherapy, antibiotic therapy, ocular candidiasis, endocarditis or thrombophlebitis, intensive care unit admission.

^*c*^ Dash indicates variables not selected by the procedure.

^*d*^ Within the prior 30 days.

^*e*^ Within the prior 90 days.

^*f*^ 52 subjects (12 deaths).

^*g*^ 60 subjects (13 deaths).

^*h*^ 59 subjects (13 deaths).

^*i*^ Abbreviations: aHR: adjusted hazard ratio; BC: blood cultures; BSI: bloodstream infection; CI: confidence interval; D: days; HR: hazard ratio; IQR: interquartile range; Y: years; FLZR-CP BSI: fluconazole-resistant *C. parapsilosis* bloodstream infection; L-AmB: liposomal amphothericin B.

^*j*^ Empty cells indicate variables for which multivariate analysis was not performed.

**TABLE 3:** Shared frailty analysis and landmark analysis (5 days) of multivariable with backward selection analysis of factors associated with 30-day all-cause mortality in the study population, after missing imputation^*a*^^,^^*b*^

	Shared frailty analysis		Landmark analysis	
Variable	aHR (95% CI)	*P*-value	aHR (95% CI)	*P*-value
Treatment (echinocandin vs L-AmB)	0.71 (0.24–2.08)	0.5338	0.79 (0.26–3.39)	0.6782
Diabetes mellitus (yes vs no)	4.92 (1.36–17.86)	**0.0153**	3.47 (0.92–13.00)	0.0655
Chronic lung disease (yes vs no)	6.76 (1.67–27.34)	**0.0073**	5.95 (1.55–22.80)	**0.0092**
Septic shock (yes vs no)	7.18 (2.17–23.70)	**0.0012**	6.33 (2.00–20.04)	**0.0017**
Source control done (yes vs no)	0.12 (0.02–0.66)	**0.0153**	0.25 (0.03–2.26)	0.2185

^*a*^ Analyses conducted after multiple imputation. The reported *P*-values are from the Cox regression analysis. Bold values are significant at the selected level of significance (α = 0.05).

^*b*^ Abbreviations: aHR: adjusted hazard ratio; CI: confidence interval.

### Analysis of the impact of targeted therapy on the rate of persistent FLZR-CP BSI

Overall, 60 out of 63 (95.2%) patients had at least one set of follow-up blood cultures. According to our study definition, persistent candidemia was detected in 22 of 60 (36.7%) patients. This included 15 of 41 patients (36.6%) treated with echinocandins and 7 of 19 patients (36.8%) treated with L-AmB. Univariate and multivariate analyses evaluating the association between echinocandins, L-AmB, and persistent candidemia are reported in the Table S1 and S2. The type of targeted therapy was not significantly associated with persistent FLZR-CP BSI (adjusted odds ratio [aOR] 1.61; 95% CI 0.43–5.99, *P* = 0.4764). Consistent findings were also observed in the additional model adjusted for clustering (aOR 1.61; 0.48–5.47, *P* = 0.4249; data not shown).

## DISCUSSION

To our knowledge, this is the first study to compare the outcomes of targeted antifungal therapy with echinocandins and L-AmB in the treatment of FLZR-CP BSI. No significant differences were observed in our study between groups in terms of 30-day all-cause mortality or persistent candidemia. In multivariable analysis, independent predictors of 30-day all-cause mortality included diabetes mellitus and chronic lung diseases. Septic shock at presentation was also a strong predictor of mortality, whereas adequate source control was protective. The choice of targeted antifungal therapy did not impact mortality.

In recent years, the use of echinocandins to treat FLZS-CP BSI has become increasingly common in clinical practice (22). Despite theoretical concerns related to a higher MIC for echinocandins in *C. parapsilosis* compared to other *Candida* species (9, 10), there are robust data supporting this practice in patients with FLZS-CP (23–29). Indeed, there have been four randomized controlled trials (23–26) and one meta-analysis (30) evaluating the use of echinocandins for the treatment of adult patients with invasive candidiasis due to *C. parapsilosis.* All of these showed no statistically significant differences in clinical response rates for the echinocandins versus comparators. There have also been several high-quality retrospective cohort studies that have yielded similar results (28, 29). A large multicenter study by Fernández-Ruiz et al. (28) revealed no significant difference in all-cause mortality in patients with FLZS-CP who were initially treated (within 72 hours) with echinocandins. Chiotos et al. (29) retrospectively evaluated the efficacy of echinocandins in comparison to fluconazole as targeted antifungal therapy for FLZS-CP and similarly found no differences in terms of outcomes between groups.

While the efficacy of echinocandins in FLZS-CP BSI is well established based on these studies (23–29), their role in treating FLZR-CP BSI remains unexamined. Our study addresses this gap by exclusively including patients with FLZR-CP BSI, offering further evidence on the therapeutic efficacy of echinocandins in this setting. Notably, our findings might be particularly relevant given the increasing prevalence of FLZR-CP across various geographic regions (1, 2), with some studies reporting fluconazole resistance rates as high as 80% (31–33). Moreover, unlike previous studies where early intravascular device removal may have influenced antifungal treatment selection and clinical outcomes (28), our results remained consistent even in the subgroup of patients with FLZR-CP BSI (*n* = 11) for whom adequate source control was not performed.

Persistent candidemia has traditionally been reported in approximately 20%–25% of patients with candidemia (34, 35) and is associated with onco-hematological disease, neutropenia, biofilm formation, inadequate antifungal therapy, and delayed or incomplete source control (36). In our study, in which follow-up blood cultures were available for 95.2% of cases, the rate of persistent candidemia (~30%) was consistent with previous reports. We were not able to demonstrate any significant difference in the rate of persistent FLZR-CP BSI between patients treated with echinocandins and those receiving L-AmB. This finding contrasts with two previous randomized trials that reported a higher incidence of persistent fungemia (23, 24) and a lower eradication rate (24) when FLZS-CP BSI were treated with echinocandins. However, differences in study design, local epidemiology (23), and baseline patient characteristics may explain these discrepancies. Regardless of the targeted antifungal therapy administered, our study reinforces the importance of early identification and appropriate management of the infection source in *Candida* BSI (37).

Our study has several limitations, and some findings should be interpreted with caution. First, we acknowledge that the choice of antifungal therapy was determined by the treating clinicians and may have been influenced by patient characteristics, introducing selection bias and potential confounding. While no method can fully adjust for differences between patients receiving echinocandins or L-AmB, we attempted to minimize these variations by matching patients based on age, key severity factors, infection source, and time-to-treatment initiation. Nonetheless, measured confounding may still have influenced treatment allocation and outcomes. Second, the analysis was limited by the small number of patients treated with L-AmB, leading to wide CIs. Although this represents the largest study available to date, further studies are needed to validate our findings. Third, few cases of ocular candidiasis were included, and ophthalmologic examination was performed in about half of the patients (38). Consequently, these results cannot be generalized to FLZR-CP BSI associated with ocular candidiasis, where echinocandins have limited efficacy due to poor ocular penetration (11). Fourth, we did not collect data regarding echinocandins MIC. Therefore, we were unable to explore the relationship between clinical failure and echinocandins MIC values. Further studies addressing this aspect are warranted. Fifth, although the time from blood culture collection to initiation of targeted antifungal therapy was collected and was similar between groups, the exact time from microbiological identification (i.e., blood culture positivity) to treatment initiation was not available, which may represent a minor limitation in assessing early treatment dynamics. Sixth, we lacked standardized daily follow-up blood cultures. Although >90% of patients had at least one follow-up blood culture within 5 days, the absence of systematic daily sampling may have led to misclassification of persistent candidemia. While variability in the timing of antifungal initiation and source control can influence candidemia clearance and thus the secondary outcome, these variables did not significantly differ between treatment groups in our cohort. Seventh, long-term outcomes in patients with FLZR-CP BSI were not assessed. Evaluating outcomes such as late recurrent candidemia (39) and 1-year mortality (19) could provide additional insights and represent an important avenue for future research. Lastly, data on acute kidney injury was not systematically collected, despite its recognized association with L-AmB therapy. However, our study was not designed to assess this specific complication.

In conclusion, we were not able to detect any differences in 30-day all-cause mortality rates or persistent fungemia between patients with FLZR-CP treated with either echinocandins or L-AmB. While awaiting randomized clinical trials to confirm these findings, our data further support current guidelines recommending the use of echinocandins for the treatment of *C. parapsilosis* fungemia, including infections caused by fluconazole-resistant strains.

## SUPPLEMENTAL MATERIAL

Supplemental material aac.00355-25-s0001.docx Fig. S1 and S2; Table S1 and S2.

ASM does not own the copyrights to Supplemental Material that may be linked to, or accessed through, an article. The authors have granted ASM a non-exclusive, world-wide license to publish the Supplemental Material files. Please contact the corresponding author directly for reuse.

## References

[1] Daneshnia F, de Almeida Júnior JN, Ilkit M, Lombardi L, Perry AM, Gao M, Nobile CJ, Egger M, Perlin DS, Zhai B, Hohl TM, Gabaldón T, Colombo AL, Hoenigl M, Arastehfar A. 2023. Worldwide emergence of fluconazole-resistant Candida parapsilosis: current framework and future research roadmap. Lancet Microbe 4:e470–e480. doi:10.1016/S2666-5247(23)00067-8 37121240 PMC10634418

[2] Escribano P, Guinea J. 2022. Fluconazole-resistant Candida parapsilosis: a new emerging threat in the fungi arena. Front Fungal Biol 3:1010782. doi:10.3389/ffunb.2022.1010782 37746202 PMC10512360

[3] Vena A, Tiseo G, Falcone M, Bartalucci C, Marelli C, Cesaretti M, Di Pilato V, Escribano P, Forniti A, Giacobbe DR, Guinea J, Limongelli A, Lupetti A, Machado M, Mikulska M, Salmanton-García J, Soriano-Martin A, Taramasso L, Valerio M, Bouza E, Muñoz P, Bassetti M. 2025. Impact of fluconazole resistance on the outcomes of patients with Candida parapsilosis bloodstream infections: a retrospective multicenter study. Clin Infect Dis 80:540–550. doi:10.1093/cid/ciae603 39810592

[4] Mesini A, Mikulska M, Giacobbe DR, Del Puente F, Gandolfo N, Codda G, Orsi A, Tassinari F, Beltramini S, Marchese A, Icardi G, Del Bono V, Viscoli C. 2020. Changing epidemiology of candidaemia: increase in fluconazole-resistant Candida parapsilosis. Mycoses 63:361–368. doi:10.1111/myc.13050 31954083

[5] Thomaz DY, De Almeida JN, Lima GME, Nunes MDO, Camargo CH, Grenfell RDC, Benard G, Del Negro GMB. 2018. An azole-resistant Candida parapsilosis outbreak: clonal persistence in the intensive care unit of a Brazilian teaching hospital. Front Microbiol 9:2997. doi:10.3389/fmicb.2018.02997 30568646 PMC6290035

[6] Pappas PG, Kauffman CA, Andes DR, Clancy CJ, Marr KA, Ostrosky-Zeichner L, Reboli AC, Schuster MG, Vazquez JA, Walsh TJ, Zaoutis TE, Sobel JD. 2016. Clinical practice guideline for the management of candidiasis: 2016 update by the infectious diseases society of America. Clin Infect Dis 62:e1–50. doi:10.1093/cid/civ933 26679628 PMC4725385

[7] Chen S-A, Perfect J, Colombo AL, Cornely OA, Groll AH, Seidel D, Albus K, de Almedia JN, Garcia-Effron G, Gilroy N, et al.. 2021. Global guideline for the diagnosis and management of rare yeast infections: an initiative of the ECMM in cooperation with ISHAM and ASM. Lancet Infect Dis 21:e375–e386. doi:10.1016/S1473-3099(21)00203-6 34419208

[8] Cornely OA, Sprute R, Bassetti M, Chen SC-A, Groll AH, Kurzai O, Lass-Flörl C, Ostrosky-Zeichner L, Rautemaa-Richardson R, Revathi G, et al.. 2025. Global guideline for the diagnosis and management of candidiasis: an initiative of the ECMM in cooperation with ISHAM and ASM. Lancet Infect Dis 25:e280–e293. doi:10.1016/S1473-3099(24)00749-7 39956121

[9] Perlin DS. 2015. Echinocandin resistance in Candida. Clin Infect Dis 61:S612–S617. doi:10.1093/cid/civ791 26567278 PMC4643482

[10] Garcia-Effron G, Katiyar SK, Park S, Edlind TD, Perlin DS. 2008. A naturally occurring proline-to-alanine amino acid change in Fks1p in Candida parapsilosis, Candida orthopsilosis, and Candida metapsilosis accounts for reduced echinocandin susceptibility. Antimicrob Agents Chemother 52:2305–2312. doi:10.1128/AAC.00262-08 18443110 PMC2443908

[11] Felton T, Troke PF, Hope WW. 2014. Tissue penetration of antifungal agents. Clin Microbiol Rev 27:68–88. doi:10.1128/CMR.00046-13 24396137 PMC3910906

[12] Ning Y, Xiao M, Perlin DS, Zhao Y, Lu M, Li Y, Luo Z, Dai R, Li S, Xu J, Liu L, He H, Liu Y, Li F, Guo Y, Chen Z, Xu Y, Sun T, Zhang L. 2023. Decreased echinocandin susceptibility in Candida parapsilosis causing candidemia and emergence of a pan-echinocandin resistant case in China. Emerg Microbes Infect 12:2153086. doi:10.1080/22221751.2022.2153086 36440795 PMC9793909

[13] Ben-Ami R, Kontoyiannis DP. 2021. Resistance to antifungal drugs. Infect Dis Clin North Am 35:279–311. doi:10.1016/j.idc.2021.03.003 34016279

[14] Safdar A, Ma J, Saliba F, Dupont B, Wingard JR, Hachem RY, Mattiuzzi GN, Chandrasekar PH, Kontoyiannis DP, Rolston KV, Walsh TJ, Champlin RE, Raad II. 2010. Drug-induced nephrotoxicity caused by amphotericin B lipid complex and liposomal amphotericin B: a review and meta-analysis. Medicine 89:236–244. doi:10.1097/MD.0b013e3181e9441b 20616663

[15] Gibbs WJ, Drew RH, Perfect JR. 2005. Liposomal amphotericin B: clinical experience and perspectives. Expert Rev Anti Infect Ther 3:167–181. doi:10.1586/14787210.3.2.167 15918775

[16] Brüggemann RJ, Jensen GM, Lass-Flörl C. 2022. Liposomal amphotericin B-the past. J Antimicrob Chemother 77:ii3–ii10. doi:10.1093/jac/dkac351 36426673 PMC9693798

[17] Stuart EA. 2010. Matching methods for causal inference: a review and a look forward. Statist Sci 25:1–21. doi:10.1214/09-STS313 PMC2943670 20871802

[18] Singer M, Deutschman CS, Seymour CW, Shankar-Hari M, Annane D, Bauer M, Bellomo R, Bernard GR, Chiche J-D, Coopersmith CM, Hotchkiss RS, Levy MM, Marshall JC, Martin GS, Opal SM, Rubenfeld GD, Poll T, Vincent J-L, Angus DC. 2016. The third international consensus definitions for sepsis and septic shock (Sepsis-3). JAMA 315:801. doi:10.1001/jama.2016.0287 26903338 PMC4968574

[19] Vena A, Bovis F, Tutino S, Santagostino Barbone A, Mezzogori L, Ponzano M, Taramasso L, Baldi F, Dettori S, Labate L, et al.. 2023. Short course of antifungal therapy in patients with uncomplicated Candida bloodstream infection: another case of less is more in the clinical setting? Open Forum Infect Dis 10:ofac656. doi:10.1093/ofid/ofac656 36655192 PMC9835756

[20] Yuan YC. 2010. Multiple imputation for missing data: concepts and new development (Version 9.0). Available from: https://facweb.cdm.depaul.edu/sjost/csc423/ documents/multipleimputation.pdf. Retrieved 14 Feb 2025.

[21] Balan TA, Putter H. 2020. A tutorial on frailty models. Stat Methods Med Res 29:3424–3454. doi:10.1177/0962280220921889 32466712 PMC7534210

[22] Hoenigl M, Salmanton-García J, Egger M, Gangneux J-P, Bicanic T, Arikan-Akdagli S, Alastruey-Izquierdo A, Klimko N, Barac A, Özenci V, et al.. 2023. Guideline adherence and survival of patients with candidaemia in Europe: results from the ECMM Candida III multinational European observational cohort study. Lancet Infect Dis 23:751–761. doi:10.1016/S1473-3099(22)00872-6 37254300

[23] Mora-Duarte J, Betts R, Rotstein C, Colombo AL, Thompson-Moya L, Smietana J, Lupinacci R, Sable C, Kartsonis N, Perfect J, Caspofungin Invasive Candidiasis Study Group. 2002. Comparison of caspofungin and amphotericin B for invasive candidiasis. N Engl J Med 347:2020–2029. doi:10.1056/NEJMoa021585 12490683

[24] Reboli AC, Rotstein C, Pappas PG, Chapman SW, Kett DH, Kumar D, Betts R, Wible M, Goldstein BP, Schranz J, Krause DS, Walsh TJ, Anidulafungin Study Group. 2007. Anidulafungin versus fluconazole for invasive candidiasis. N Engl J Med 356:2472–2482. doi:10.1056/NEJMoa066906 17568028

[25] Kuse E-R, Chetchotisakd P, Da Cunha CA, Ruhnke M, Barrios C, Raghunadharao D, Sekhon JS, Freire A, Ramasubramanian V, Demeyer I, Nucci M, Leelarasamee A, Jacobs F, Decruyenaere J, Pittet D, Ullmann AJ, Ostrosky-Zeichner L, Lortholary O, Koblinger S, Diekmann-Berndt H, Cornely OA. 2007. Micafungin versus liposomal amphotericin B for candidaemia and invasive candidosis: a phase III randomised double-blind trial. The Lancet 369:1519–1527. doi:10.1016/S0140-6736(07)60605-9 17482982

[26] Colombo AL, Perfect J, DiNubile M, Bartizal K, Motyl M, Hicks P, Lupinacci R, Sable C, Kartsonis N. 2003. Global distribution and outcomes for Candida species causing invasive candidiasis: results from an international randomized double-blind study of caspofungin versus amphotericin B for the treatment of invasive candidiasis. Eur J Clin Microbiol Infect Dis 22:470–474. doi:10.1007/s10096-003-0973-8 12884068

[27] Pappas PG, Rotstein CMF, Betts RF, Nucci M, Talwar D, De Waele JJ, Vazquez JA, Dupont BF, Horn DL, Ostrosky-Zeichner L, Reboli AC, Suh B, Digumarti R, Wu C, Kovanda LL, Arnold LJ, Buell DN. 2007. Micafungin versus caspofungin for treatment of candidemia and other forms of invasive candidiasis. Clin Infect Dis 45:883–893. doi:10.1086/520980 17806055

[28] Fernández-Ruiz M, Aguado JM, Almirante B, Lora-Pablos D, Padilla B, Puig-Asensio M, Montejo M, García-Rodríguez J, Pemán J, Ruiz Pérez de Pipaón M, Cuenca-Estrella M, et al.. 2014. Initial use of echinocandins does not negatively influence outcome in Candida parapsilosis bloodstream infection: a propensity score analysis. Clin Infect Dis 58:1413–1421. doi:10.1093/cid/ciu158 24642553

[29] Chiotos K, Vendetti N, Zaoutis TE, Baddley J, Ostrosky-Zeichner L, Pappas P, Fisher BT. 2016. Comparative effectiveness of echinocandins versus fluconazole therapy for the treatment of adult candidaemia due to Candida parapsilosis: a retrospective observational cohort study of the Mycoses Study Group (MSG-12). J Antimicrob Chemother 71:3536–3539. doi:10.1093/jac/dkw305 27494929 PMC5181395

[30] Kale-Pradhan PB, Morgan G, Wilhelm SM, Johnson LB. 2010. Comparative efficacy of echinocandins and nonechinocandins for the treatment of Candida parapsilosis Infections: a meta-analysis. Pharmacotherapy 30:1207–1213. doi:10.1592/phco.30.12.1207 21114387

[31] Magobo RE, Lockhart SR, Govender NP. 2020. Fluconazole‐resistant Candida parapsilosis strains with a Y132F substitution in the ERG11 gene causing invasive infections in a neonatal unit, South Africa. Mycoses 63:471–477. doi:10.1111/myc.13070 32124485 PMC11973574

[32] Prigitano A, Blasi E, Calabrò M, Cavanna C, Cornetta M, Farina C, Grancini A, Innocenti P, Lo Cascio G, Nicola L, Trovato L, Cogliati M, Esposto MC, Tortorano AM, Romanò L, on behalf of the FiCoV Study Group. 2023. Yeast bloodstream infections in the COVID-19 patient: a multicenter Italian study (FiCoV study). J Fungi 9:277. doi:10.3390/jof9020277 PMC9962415 36836391

[33] Thomaz DY, de Almeida JN, Sejas ONE, Del Negro GMB, Carvalho GOMH, Gimenes VMF, de Souza MEB, Arastehfar A, Camargo CH, Motta AL, Rossi F, Perlin DS, Freire MP, Abdala E, Benard G. 2021. Environmental clonal spread of azole-resistant Candida parapsilosis with Erg11-Y132F mutation causing a large candidemia outbreak in a Brazilian cancer referral center. J Fungi 7:259. doi:10.3390/jof7040259 PMC8066986 33808442

[34] Kullberg BJ, Sobel JD, Ruhnke M, Pappas PG, Viscoli C, Rex JH, Cleary JD, Rubinstein E, Church LWP, Brown JM, Schlamm HT, Oborska IT, Hilton F, Hodges MR. 2005. Voriconazole versus a regimen of amphotericin B followed by fluconazole for candidaemia in non-neutropenic patients: a randomised non-inferiority trial. The Lancet 366:1435–1442. doi:10.1016/S0140-6736(05)67490-9 16243088

[35] Colombo AL, Ngai AL, Bourque M, Bradshaw SK, Strohmaier KM, Taylor AF, Lupinacci RJ, Kartsonis NA. 2010. Caspofungin use in patients with invasive candidiasis caused by common non-albicans Candida species: review of the caspofungin database. Antimicrob Agents Chemother 54:1864–1871. doi:10.1128/AAC.00911-09 20231388 PMC2863639

[36] Agnelli C, Valerio M, Bouza E, Vena A, Guinea J, del Carmen Martínez-Jiménez M, Marcos-Zambrano LJ, Escribano P, Muñoz P, on behalf of the COMIC Study Group (Collaborative Group on Mycosis). 2019. Persistent Candidemia in adults: underlying causes and clinical significance in the antifungal stewardship era. Eur J Clin Microbiol Infect Dis 38:607–614. doi:10.1007/s10096-019-03477-3 30680572

[37] Vena A, Bouza E, Corisco R, Machado M, Valerio M, Sánchez C, Muñoz P, COMIC Study Group. 2020. Efficacy of a “checklist” intervention bundle on the clinical outcome of patients with Candida bloodstream infections: a quasi-experimental pre-post study. Infect Dis Ther 9:119–135. doi:10.1007/s40121-020-00281-x 32020522 PMC7054590

[38] Vena A, Muñoz P, Padilla B, Valerio M, Sanchez MI, Puig-Asensio M, Fortun J, Fernandez-Ruiz M, Merino P, Losa JE, Loza A, Rivas RA, Bouza E, for the CANDIPOP Project, GEIH-GEMICOMED (SEIMC), and REIPI. 2017. Is routine ophthalmoscopy really necessary in candidemic patients? PLoS One 12:e0183485. doi:10.1371/journal.pone.0183485 29065121 PMC5655487

[39] Muñoz P, Vena A, Valerio M, Álvarez-Uría A, Guinea J, Escribano P, Bouza E. 2016. Risk factors for late recurrent candidaemia. a retrospective matched case–control study. Clin Microbiol Infect 22:277. doi:10.1016/j.cmi.2015.10.023 26548507

