# Supplementary material for: Comparative effectiveness of echinocandins and liposomal amphotericin B for fluconazole-resistant Candida parapsilosis bloodstream infections
Source: Antimicrob Agents Chemother. 2025 Sep 30;69(11):e00355-25. doi: 10.1128/aac.00355-25 (PMC12587536; doi:10.1128/aac.00355-25)
Supplement: Supplemental material — Fig. S1 and S2; Table S1 and S2. [file aac.00355-25-s0001.docx]

**Supplementary Materials**

**Figure S1. Unadjusted cumulative survival probability distribution up to day 30 in patients with FLZR-CP BSI who did not receive source control (N=11) treated with echinocandins or L-AmB.**

**
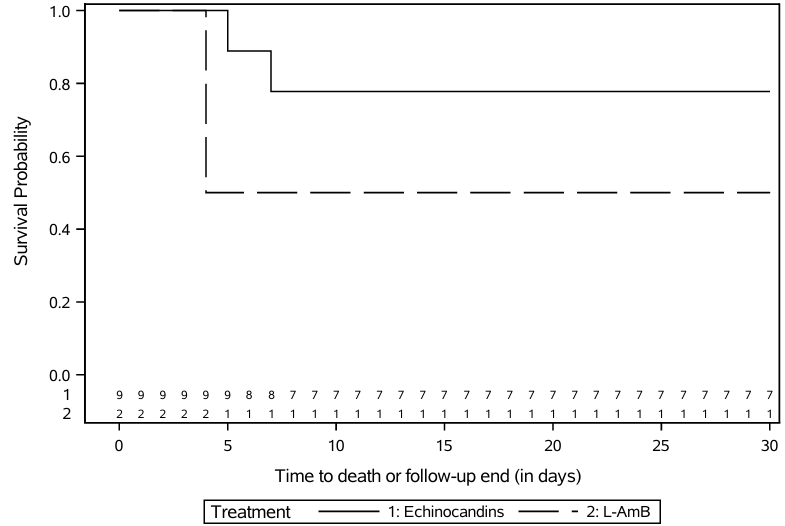
**

Sensitivity analysis: The estimated association with 30-days all-cause mortality and definitive therapy with echinocandins or L-AmB in patients who did not undergo adequate source control (n=11, 9 out of 11 [81.8%] treated with echinocandins and 2 out of 11 [18.2%] treated with L-AmB) showed no statically significant differences between the two groups (Fisher test, p=.4909)

**Figure S2. Unadjusted cumulative survival probability distribution up to day 30 in patients with FLZR-CP BSI initially treated with echinocandins or L-AmB (N=60)**

***
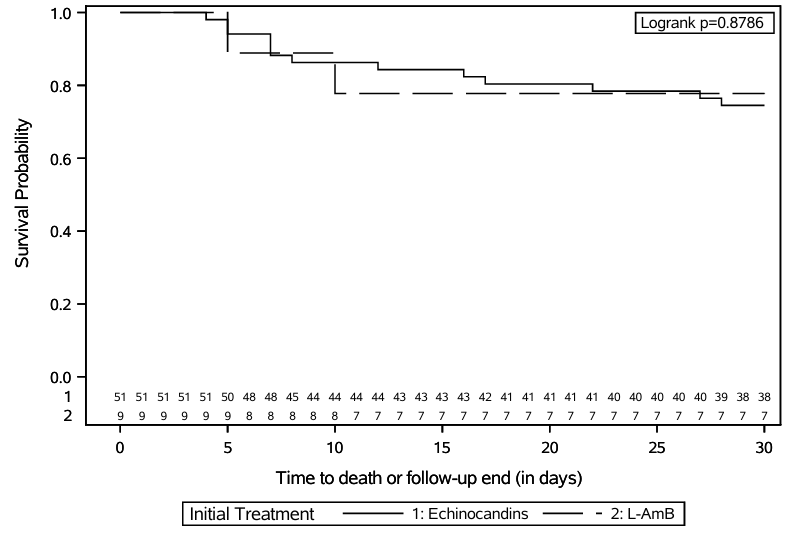
***

**Table S1.** Baseline Demographic and Clinical Characteristics of Patients with FLZR-CP BSI who had Follow-Up Blood Cultures Available (N=60), Stratified by Treatment With Echinocandins And L-AmB

| **Variables** | **Not-persistent fungemia**  **N=38** | **Persistent fungemia**  **N=22** | **p-value** |
| --- | --- | --- | --- |
| Age, median [IQR], y | 66 (58-72) | 62.5 (60-70) | 0.6450 |
| Male sex; n (%) | 22 (57.9) | 9 (40.9) | 0.2045 |
| **Hospital ward stay at the time of *C. parapsilosis* BSI; n (%)** |  |  | 0.5144^F^ |
| Intensive care unit | 26 (68.4) | 12 (54.6) |  |
| Surgical ward | 7 (18.4) | 5 (22.7) |  |
| Internal medicine ward | 5 (13.2) | 5 (22.7) |  |
| **Time between hospital admission and first positive BC; median [IQR], d** | 38 (21-66) | 35 (21-53) | 0.5496 |
| **Charlson Comorbidity Index; median (IQR)** | 2.5 (1-3) | 2.5 (1-3) | 0.6593 |
| **Underlying conditions, n (%)** |  |  |  |
| Cardiovascular disease | 13 (34.2) | 13 (59.1) | 0.0609 |
| Gastrointestinal disease | 11 (29.0) | 5 (22.7) | 0.5996 |
| Chronic lung disease | 8 (21.1) | 0 (0.0) | **0.0218^F^** |
| Solid tumour | 7 (18.4) | 8 (36.4) | 0.1219 |
| Neurological disease | 5 (13.2) | 3 (13.6) | 1.0000^F^ |
| Chronic kidney disease | 4 (10.5) | 4 (18.2) | 0.4486^F^ |
| Diabetes mellitus | 4 (10.5) | 5 (22.7) | 0.2668^F^ |
| Chronic liver disease | 2 (5.3) | 3 (13.6) | 0.3459^F^ |
| Solid organ transplantation | 2/38 (5.3) | 1/21 (4.8) | 1.0000^F^ |
| Haematological malignancy | 1 (2.6) | 1 (4.6) | 1.0000^F^ |
| **Risk factors for candidemia, n (%)** |  |  |  |
| Antibiotic therapy* | 35 (92.1) | 20 (90.9) | 1.0000^F^ |
| Central venous catheter | 28 (73.7) | 17 (77.3) | 0.7571 |
| Surgery (all types) ** | 24 (63.2) | 14 (63.6) | 0.9704 |
| Total parenteral nutrition* | 19/36 (52.8) | 13/21 (61.9) | 0.5030 |
| Corticosteroid therapy* | 18/38 (47.4) | 6/21 (28.6) | 0.1593 |
| Intra-abdominal surgery** | 12/38 (31.6) | 6/21 (28.6) | 0.8102 |
| Haemodialysis** | 10 (26.3) | 4 (18.2) | 0.4728 |
| Immunosuppressive therapy* | 6/37 (16.2) | 1/22 (4.6) | 0.2401^F^ |
| Chemotherapy** | 1 (2.6) | 1 (4.6) | 1.0000^F^ |
| **Previous antifungal treatment, n (%)*** | 9/38 (23.7) | 8/21 (38.1) | 0.2419 |
| **Time between first positive BC and appropriate initial antifungal therapy^#^, median [IQR], d** | 2 (1-3) | 3 (1-4) | **0.0334** |
| **Septic shock^#^, n (%)** | 7 (18.4) | 5 (22.7) | 0.7444^F^ |
| **Primary/catheter source of origin (vs. abdominal); n (%)** | 36 (94.7) | 19 (86.4) | 0.3459^F^ |
| **Source control done; n (%)** |  |  | 0.8561^F^ |
| Yes | 30/36 (83.3) | 19/22 (86.4) |  |
| No | 1/36 (2.8) | 1/22 (4.6) |  |
| Not possible | 5 /36 (13.9) | 2/22 (9.1) |  |
| **Time between first positive BCs and source control, median [IQR], d** | n=28  2 (1-3) | n=19  3 (2-6) | **0.0342** |
| Treatment (echinocandin vs. L-AmB) | 26 (68.4) | 15 (68.2) | 0.9847 |
| **Diagnostic procedures, n (%)** |  |  |  |
| Echocardiography and echocolordoppler | 35 (92.1) | 21 (95.5) | 1.0000^F^ |
| Ophthalmologic examination | 22 (57.9) | 15 (68.2) | 0.4297 |
| **Complications, n (%)** |  |  |  |
| Ocular candidiasis |  |  | 0.3852^F^ |
| No | 22 (57.9) | 14 (63.6) |  |
| Yes | 0 (0.0) | 1 (4.6) |  |
| Endocarditis or thrombophlebitis |  |  | 0.7563^F^ |
| No | 32 (84.2) | 18 (81.8) |  |
| Yes | 3 (7.9) | 3 (13.6) |  |
| Intensive care unit admission | 2 (5.3) | 3 (13.6) | 0.3459^F^ |
| Need for haemodialysis after *C.parapsilosis* BSI | 4/37 (10.8) | 4/22 (18.2) | 0.4551^F^ |
| **30-d all-cause mortality, n (%)** | 9 (23.7) | 4 (18.2) | 0.7508^F^ |
| **Time between first positive BCs and death, median [IQR], d** | n=14  9 (7-51) | n=7  28 (22-32) | 0.2170 |

The reported p-values are from the Wilcoxon Rank Sum test for continuous variables and Chi-Square or Fisher’s Exact test for categorical variables;

Bold values are significant at the selected level of significance (α = 0.05).

Where indicated, due to missing values, the analysis is based on the available sample size.

^#^ Variable transformed for matching purposes and differs from the categorization presented in this table.

* within the prior 30 days.

******** within the prior 90 days.

^F^ Fisher’s Exact test.

**Abbreviations**: **BC**: Blood cultures; **BSI:** Bloodstream infection; **D**: days; **IQR:** Interquartile range; **L-AmB**: liposomial amphothericin B; **Y**: years**.**

**Table S2.** Multivariable Analyses of Factors Associated with Persistent FLZR-CP BSI in the Study Population, After Missing Imputation

|  | **Multivariable analysis***** | |
| --- | --- | --- |
| **Variable** | **aOR (95% CI)** | **p-value** |
| Treatment (echinocandin vs. L-AmB)* | 1.61 (0.43-5.99) | 0.4764 |
| **Time between first positive BCs and source control**, d** | 1.40 (1.01-1.94) | **0.0427** |

Analyses were conducted after multiple imputation and stepwise backward selection. Reported p-values are derived from logistic regression analysis. Bold values indicate statistical significance at the predefined α level of 0.05. Multicollinearity absence and model goodness-of-fit were verified.

The variable Source control done was excluded from the analysis due to the inclusion of Time between first positive blood culture and source control as a covariate.

* Variable not selected by the stepwise backward procedure (p=0.9144) but forced into the model.

** In the stepwise backward selection, this variable was initially categorized using the Source control done variable to account for missing values. After being selected, it was treated as a continuous variable (as in Table 1) to assess the impact of a one-day change on the outcome, rather than considering categorical distinctions.

*** 51 subjects, 19 with persistent positive blood cultures.

**Abbreviations:** **aOR:** Adjusted Odds Ratio; **BC**: Blood cultures; **L-AmB**: Liposomial amphothericin B; **CI:** Confidence Interval; **D**: days; **OR**: Odds Ratio.
